# AN INTERGENERATIONAL TRAINING PROGRAM TO SUPPORT LATINE ELDERS: LESSONS LEARNED

**DOI:** 10.1093/geroni/igad104.0155

**Published:** 2023-12-21

**Authors:** Quinton Cotton, Paul Kissell, Elma Johnson, Yolima Chambers, Manka Nkimbeng, Joseph Gaugler

**Affiliations:** University of Minnesota, Minneapolis, Minnesota, United States; University of Minnesota School of Public Health, Minneapolis, Minnesota, United States; University of Minnesota, Minneapolis, Minnesota, United States; Centro Tyrone Guzman, Minneapolis, Minnesota, United States; University of Minnesota, Minneapolis, Minnesota, United States; University of Minnesota, Minneapolis, Minnesota, United States

## Abstract

Cultural adaptation and tailoring of caregiving interventions has garnered greater attention in part due to high levels of unmet need and poor intervention outcomes among dementia caregivers from racial and cultural groups. Additionally, there is a growing interest in understanding intervention mechanisms. We conducted a formative analysis of the process employed in the design of an intergenerational training program to support elders within the Latine community. A content analysis of memos and stakeholder listening sessions and document review guided this analysis. Framed within a discussion on the continuum of cultural adaptation, we report on lessons learned. Lessons include: 1) embracing community-led design, 2) demonstration of partnership commitment to facilitate trust-building, 3) consistent appreciative inquiry, and 4) demonstration of cultural centeredness within the partnership.

